# Research on Two-Stream Networks Integrating Physiological Features and Attention Mechanisms for Motion Classification in Visually Impaired Individuals

**DOI:** 10.3390/s26123681

**Published:** 2026-06-09

**Authors:** Wentong Wang, Changyuan Wang, Zehui Chen, Wenbo Huang

**Affiliations:** 1 School of Optoelectronic Engineering, Xi’an Technological University, Xi’an 710021, China; wangwentong@st.xatu.edu.cn (W.W.); chenzehui@st.xatu.edu.cn (Z.C.); 2 Mechanical Manufacturing and Automation Program, Shaanxi Polytechnic University, Xianyang 712000, China; 20231748@sxpi.edu.cn

**Keywords:** assistive mobility for the blindfolded mobility simulation, multi-modal data fusion, deep learning, attention mechanism, motion classification

## Abstract

To address the issues of low perception accuracy and poor robustness in traditional motion recognition methods within complex walking environments for visually impaired individuals, this study utilizes multi-modal data, including ECG, PPG, and IMU, for classification. Regarding the low filtering efficiency of multi-modal data, an improved wavelet filtering algorithm based on LSTM is proposed. To further enhance classification accuracy, this paper introduces a motion recognition method for the blindfolded mobility simulation based on an Attention-based Two-Stream Deep Fusion Convolutional Neural Network (ATS-DFCNN). The proposed method constructs a two-stream heterogeneous feature extraction architecture by synchronously collecting tri-axial motion signals and physiological signals from subjects. A 1D-CNN is employed to capture the spatial geometric features of limb movements, while a hybrid CNN-GRU network is utilized to mine the temporal evolution patterns of physiological stress. Furthermore, an attention mechanism is introduced to achieve dynamic weighted fusion at the feature level, which strengthens critical motion features and suppresses environmental noise. Experiments were conducted with 10 subjects simulating the movements of visually impaired individuals, covering typical actions such as walking, standing, climbing stairs, descending stairs, and falling. The results demonstrate that the proposed adaptive filtering algorithm achieves an AUC of 0.942, significantly improving feature distinctiveness compared to traditional algorithms. The ATS-DFCNN model achieved an average recognition accuracy of 92.2% across five activity categories, representing a 4.8% performance increase over single IMU modal classification. Particularly in fall detection, the model effectively reduces false alarms through physiological feedback and accurately infers motion intentions, providing reliable technical support for the safety monitoring of intelligent walking-aid systems.

## 1. Introduction

With the continuous advancement of assistive technologies [1], enhancing the autonomy and safety of visually impaired individuals during daily travel has become a vital research direction in intelligent perception and assistive engineering [2]. Due to the lack of visual information, these individuals are highly susceptible to safety accidents such as collisions, trips, and falls in complex environments. The World Health Organization reports that hundreds of millions of people globally live with visual impairments [3]. A significant portion of this population faces restricted mobility because they lack effective motion perception and assistive systems [4].

The core challenge in assistive technology research for the visually impaired lies in accurately perceiving user motion states and contexts within dynamic, complex, and highly uncertain real-world environments [5]. A major obstacle in motion classification and fall detection is the accurate recognition of atypical gait behaviors [6]. Specifically, the movement patterns of the visually impaired differ significantly from those of sighted individuals when using white canes, hesitating, or facing sudden environmental changes. Furthermore, certain behaviors share similar kinematic characteristics but differ fundamentally in risk levels and psychophysiological states, such as “active stopping” versus “emergency stopping due to obstacles.” Traditional methods relying solely on single inertial sensors or vision data struggle to distinguish these complex behaviors, leading to insufficient accuracy and high false-alarm rates in real-world scenarios [7].

Human activity recognition (HAR) systems based on sensor data are widely studied in healthcare and intelligent mobility. Vision-based methods identify specific actions by extracting spatio-temporal features from skeletal keypoint information via cameras [8]. However, these methods are sensitive to lighting, occlusion, and camera placement, and they face inherent limitations in privacy protection, making them unsuitable for all-weather, wearable assistive applications [9].

In contrast, wearable inertial sensor [10] solutions based on Micro-Electro-Mechanical Systems (MEMS) have become a mainstream research direction due to their low power consumption, non-invasiveness, and environmental adaptability [11,12,13]. For instance, the DeepConvLSTM framework demonstrates high recognition precision in processing long-sequence inertial data [14,15]. Nevertheless, relying solely on inertial data fails to reflect the user’s physiological load or psychological stress [16], particularly in distinguishing non-motor physiological changes. When a visually impaired individual perceives an obstacle, their physiological signals may change significantly even if their kinematic features remain subtle.

To address these deficiencies, some studies have introduced physiological signals—such as ECG and Photoplethysmography (PPG)—as auxiliary information [17]. The Pan–Tompkins algorithm provides a classic solution for real-time QRS complex detection [18], laying the foundation for subsequent physiological feature extraction [19]. Research indicates that physiological indicators like heart rate variability and pulse wave characteristics are valuable for reflecting stress states, thereby enhancing the robustness of complex behavior recognition. Existing studies have explored classification frameworks based on fuzzy similarity to enhance model robustness against noisy and heterogeneous sensor data. By introducing a similarity modeling mechanism that accounts for tolerance uncertainty, such approaches can effectively elevate classification stability in scenarios involving complex signal interference and highly variable environments [20].

Recently, multi-modal data fusion has been recognized as a key approach to improving HAR system performance [21]. Studies on visually impaired and elderly populations show that joint modeling of inertial and physiological data significantly increases accuracy in fall detection and abnormal behavior recognition [22]. However, existing multi-modal methods often employ simple feature concatenation or decision-level fusion, lacking the ability to cooperatively model deep semantic features across different modalities. Furthermore, some deep learning models are limited by one-to-one posture mapping mechanisms [23], making it difficult to predict complex anomalies like fall precursors. Consequently, existing research has not fully resolved the issue of refined motion classification that incorporates psychophysiological load characteristics.

Based on the aforementioned analysis, this study improves upon existing wearable sensor-based methodologies. In contrast to conventional schemes that rely solely on a single kinematic signal, this work comprehensively leverages the multidimensional informational advantages of inertial posture sensors, electrocardiogram (ECG) signals, and photoplethysmogram (PPG) signals to construct a multi-modal deep feature fusion model. Through a deep neural network, joint learning is performed across different modal features to enhance the system’s perception of environmental variations and user states, thereby improving the overall accuracy and reliability of the assistive system in motion classification and fall detection tasks. Although the proposed wavelet-LSTM adaptive denoising framework exhibits excellent noise suppression capabilities, its preprocessing-related parameters are still predominantly determined through empirical tuning. Previous studies have demonstrated that relying on intelligent optimization strategies, such as the AI-driven Taguchi method, can significantly elevate the robustness and generalizability of multi-modal intelligent systems in uncertain environments [24]. Integrating AI-driven parameter optimization methods into the adaptive denoising and multi-modal fusion pipelines could further enhance data preprocessing stability, anti-noise performance, and proof-of-concept reliability in wearable sensing scenarios, thereby adapting to intelligent health monitoring applications [25].

## 2. Signal Extraction and Preprocessing

### 2.1. Physiological Signal Extraction

To extract features related to human exercise load and emotional stress from ECG and PPG signals, this study adopts a multi-dimensional feature extraction strategy. The physiological signal extraction process primarily consists of R-wave peak localization, Heart Rate Variability (HRV) analysis, and morphological feature calculation.

#### 2.1.1. ECG Signal Extraction

ECG signals contain abundant electrophysiological information, the core of which lies in detecting the QRS complex. This study employs the classic Pan-Tompkins algorithm. First, a band-pass filter (5–15 Hz) is applied to the raw signal to eliminate EMG interference. Subsequently, precise localization of R-wave peaks is achieved through derivative operations, squaring processes, and moving window integration.

Based on the extracted R-R interval (RRI) sequences, the mean heart rate (reflecting baseline exercise intensity) and the Root Mean Square of Successive Differences (RMSSD) are calculated. The RMSSD is used to reflect parasympathetic activity for identifying physiological stress after a fall. The time-domain formula is expressed as Equation (1):

(1) $$RMSSD=\sqrt{\frac{1}{N-1}\sum_{i=1}^{N-1}\;(RR_{i+1}-RR_{i}{)}^{2}}$$

#### 2.1.2. PPG Signal Extraction

Since pulse signals are highly susceptible to motion artifacts during physical activity, this study focuses on extracting waveform morphological features. The accelerated photoplethysmogram (APG) is derived using the second derivative to identify the a, b, c, d, and e waves, followed by the calculation of the waveform envelope area. Additionally, the Pulse Transit Time (PTT)—defined as the time interval between the ECG R-peak and the PPG peak within the same cardiac cycle—is calculated. This metric is highly correlated with blood pressure and autonomic nervous tension. It serves as an auxiliary indicator to assess the startle response of visually impaired individuals when encountering obstacles.

### 2.2. Motion Signal Feature Extraction

Motion signals are captured using a tri-axial accelerometer (Acc) and a tri-axial gyroscope (Gyro), which record linear acceleration and angular velocity, respectively. For the motion classification task, key features are extracted across three dimensions: time domain, frequency domain, and spatial orientation. Time-domain features reflect the intensity and rhythm of movements, while frequency-domain features are utilized to distinguish periodic actions from sudden events. To eliminate the impact of sensor orientation bias, the Signal Magnitude Vector (SMV) of the tri-axial acceleration is calculated as a representation of total motion intensity, as shown in Equation (2):

(2) $$SMV=\sqrt{a_{x}^{2}+a_{y}^{2}+a_{z}^{2}}$$

Mean, standard deviation, zero-crossing rate, and skewness are calculated within a sliding window. The mean reflects the distribution of gravitational components of the movement, while the standard deviation characterizes the intensity of the action.

The signals are converted to the frequency domain using the Fast Fourier Transform (FFT) to extract power spectral density and dominant frequency components. The dominant frequency of normal walking for the blindfolded healthy subjects typically ranges from 1.5 Hz to 2.5 Hz. In contrast, the dominant frequency for climbing stairs is slightly higher, with a more concentrated energy distribution.

### 2.3. Data Preprocessing

To eliminate discrepancies in sampling frequencies, dimensions, and environmental noise across different signal modalities, the raw data underwent refined preprocessing before feature extraction.

#### 2.3.1. Signal Synchronization and Time-Domain Resampling

In this study, the initial sampling frequencies for the Inertial Measurement Unit (IMU), ECG, and PPG sensors are $f_{IMU}$ = 100 Hz, $f_{ECG}$ = 512 Hz, and $f_{PPG}$ = 64 Hz respectively. To achieve deep feature-level fusion, these three modalities must be aligned in the time domain. Using the highest frequency ECG signal as the reference, this paper performs up-sampling on the IMU and PPG signals using clinear interpolation. Given the original discrete sampling points $\left(t_{i},y_{i}\right)$ the interpolation function S(t) on the sub-interval $\left[t_{i},t_{i+1}\right]$ is expressed as Equation (3):

(3) $$S(t)=a_{i}(t-t_{i}{)}^{3}+b_{i}(t-t_{i}{)}^{2}+c_{i}(t-t_{i})+d_{i}$$

By solving the constraint equations for second-derivative continuity, a smooth continuous signal curve is obtained and resampled at 512 Hz. This ensures that each data modality has an identical number of sampling points within the same time slice, effectively eliminating phase deviations.

Although uniform resampling to 512 Hz simplifies temporal alignment and the multi-modal fusion pipeline, it inevitably introduces interpolation redundancy for low-frequency signals. Preliminary investigations into alternative synchronization strategies indicate that a multi-rate asynchronous feature-level alignment approach could reduce computational overhead; however, it necessitates the construction of a substantially more complex temporal matching model. Consequently, during this proof-of-concept stage, a uniform synchronization scheme was selected in this study to prioritize temporal consistency and model execution stability.

#### 2.3.2. Theoretical Analysis of Wavelet Transform

Wavelet transform is a methodology that decomposes signals into wavelet functions of various scales and frequencies, providing comprehensive information in both the time and frequency domains. It employs a set of wavelet basis functions characterized by localization and multi-scale analysis properties. By adjusting scale and translation parameters, these functions enable localized analysis of the signal. Unlike the Fourier transform, which only provides global frequency information, the wavelet transform offers time-frequency localization capabilities. Consequently, it accurately reflects the transient characteristic changes of information within short time intervals.

For a square-integrable real space $L^{2}(R)$, let $\psi (t)\in L^{2}(R)$. Its Fourier transform ψ(ω)

(4) $$C_{\psi }=\int_{R}\;\frac{|\psi (\omega ){|}^{2}}{\left|\omega \right|}d\omega <\infty$$

Let ψ(t) be a mother wavelet. By translating and scaling this basis, a family of continuous wavelet sequences can be obtained:

(5) $$\psi_{a,b}(t)=|a{|}^{-1/2}\psi \left(\frac{t-b}{a}\right)$$

where a is the scale factor and b is the translation factor. For a signal f(t), the Continuous Wavelet Transform (CWT) is defined as:

(6) $$\left(\begin{array}{c} W_{\psi }f \end{array}\right)(a,b)=f,\psi_{a,b}\;=\;|a{|}^{-1/2}\int_{R}\;f(t)\psi^{*}\left(\frac{t-b}{a}\right)dt$$

where $\psi^{*}(t)$ is the complex conjugate of $\psi_{a,b}(t)$. The inverse transform is expressed as:

(7) $$f(t)=\frac{1}{C_{\psi }}\int_{-\infty }^{+\infty }\;\int_{-\infty }^{+\infty }\;\left(W_{\psi }f\right)(a,b)\psi_{a,b}(t)\frac{1}{a^{2}}dadb$$

Discretizing the CWT yields the Discrete Wavelet Transform (DWT), where the scale and translation parameters are typically represented as:

(8) $$(a,b)=\left(\begin{array}{c} a_{0}^{j},ka_{0}^{j}b_{0} \end{array}\right)j,k\in Z$$

The corresponding family of discrete wavelet functions is expressed as:

(9) $$\psi_{j,k}(t)=a_{0}^{-j/2}\psi \left(\frac{t-ka_{0}^{j}b_{0}}{a_{0}^{j}}\right)=a_{0}^{-j/2}\psi \left(a_{0}^{-j}t-kb_{0}\right)$$

The DWT coefficients for signal f(t) are given by:

(10) $$C_{j,k}=f(t),\psi_{j,k}(t)=\int_{-\infty }^{+\infty }\;f(t)\psi_{j,k}^{*}(t)dt$$

Consequently, the reconstruction formula is:

(11) $$f(t)=\sum_{-\infty }^{+\infty }\sum_{-\infty }^{+\infty }C_{j,k}\psi_{j,k}(t)$$

Commonly used thresholding functions include hard and soft thresholding. The hard thresholding function is defined as:

(12) $${\hat{\omega }}_{j,k}=\left\{\begin{array}{l} \omega_{j,k},\;\;\left|\omega_{j,k}\right|\geq \lambda \\ 0,\;\;\;\;\;\;\left|\omega_{j,k}\right|<\lambda \end{array}\right.$$

where λ is the threshold, and $\omega_{j,k}$ and ${\hat{\omega }}_{j,k}$ represent the wavelet coefficients before and after denoising, respectively. The soft thresholding function is expressed as:

(13) $${\hat{\omega }}_{j,k}=\left\{\begin{array}{l} sgn\left(\omega_{j,k}\right)\left(\left|\omega_{j,k}\right|-\lambda \right),\;\;\left|\omega_{j,k}\right|\geq \lambda \\ \;\;\;\;\;\;\;\;\;\;\;\;\;\;\;\;\;\;\;\;\;\;\;\;0,\;\;\;\;\;\;\;\;\;\;\;\;\left|\omega_{j,k}\right|<\lambda \end{array}\right.$$

Hard thresholding can preserve local information such as abrupt signal changes; however, it suffers from discontinuities, leading to oscillations in the filtered signal. While the soft thresholding function is continuous and provides smoother results, it introduces a fixed bias that may result in incomplete noise removal. Therefore, threshold selection is critical to denoising performance.

Physiological signals collected by wearable sensors are highly susceptible to motion artifacts, environmental interference, and non-stationary noise. To improve signal quality and enhance the robustness of multi-modal feature extraction, this study proposes a wavelet-LSTM adaptive denoising framework. First, the discrete wavelet transform (DWT) is utilized to decompose the raw physiological signal x(t). Multi-level wavelet decomposition can partition the signal into approximation and detail coefficients across different frequency bands:

(14) $$x(t)=A_{J}+\sum_{j=1}^{J}\;D_{j}$$

where $A_{J}$ represents the low-frequency approximation coefficients obtained from the J layer of decomposition, and $D_{j}$ denotes the high-frequency detail coefficients of the j layer.

In this study, the Daubechies wavelet (db4) was selected due to its excellent time-frequency localization properties in physiological signal processing. A 4-level wavelet decomposition strategy was adopted: the approximation coefficients $A_{4}$ were retained as the low-frequency physiological trend component, while adaptive noise suppression processing was applied to the detail coefficients $D_{1}$∼$D_{4}$.

To address the limited adaptability of fixed thresholds, we introduce an adaptive threshold adjustment algorithm based on Long Short-Term Memory (LSTM) networks. Since traditional thresholds λ are typically fixed values based on empirical data, they struggle with non-stationary noise and dynamic feature amplitudes. We propose an adaptive threshold λ = Λ(ω{j, k}, j, k; Θ), where Θ represents the learnable parameters.

(15) $$v_{x}=\frac{exp\left(c_{x}\right)}{\sum_{i=1}^{m}\;c_{i}}$$

(16) $$y_{t}=\sum_{i=1}^{t}\;v_{i}c_{i}$$

where m denotes the number of neurons, x is the neuron index, $c_{x}$ is the output value, $y_{t}$ is the input value, and $v_{x}$ represent the weights and bias, respectively.

The LSTM network is responsible for fitting the overall wavelet coefficient values by leveraging the preceding wavelet coefficients up to the current time step. Features of the wavelet coefficients are established through the input gate, forget gate, and output gate of the long short-term memory network. Finally, distinct weights are assigned to different hidden layer units of the LSTM network, yielding the final fitted output after training.

Specifically, the LSTM network takes the serialized wavelet detail coefficients as input:

(17) $$\theta_{j}=LSTM(D_{j})$$

where $\theta_{j}$ represents the adaptive threshold parameter corresponding to the j-th layer of detail coefficients.

Subsequently, soft-thresholding filtering is executed on the corresponding wavelet coefficients:

(18) $${\hat{D}}_{j}=sign(D_{j})\cdot max\left(|D_{j}|-\theta_{j},0\right)$$

where ${\hat{D}}_{j}$ represents the denoised wavelet detail coefficients.

After completing the adaptive threshold filtering, the denoised physiological signal is reconstructed via the inverse discrete wavelet transform (IDWT):

(19) $$\hat{x}(t)=IDWT(A_{J},{\hat{D}}_{j})$$

To optimize the adaptive denoising process, the mean squared error (MSE) is adopted as the objective function to train the LSTM network, minimizing the error between the reconstructed denoising signal and the reference denoising target:

(20) $$L=\frac{1}{N}\sum_{i=1}^{N}\;(x_{i}-{\hat{x}}_{i}{)}^{2}$$

where $x_{i}$ represents the reference signal sample, and ${\hat{x}}_{i}$ denotes the reconstructed denoised output.

Since completely noise-free physiological signals are extremely difficult to acquire in practical wearable sensing environments, this study utilizes conventional wavelet filtering to generate a pseudo-clean reference signal, which serves as the supervision signal during the training phase. The proposed wavelet-LSTM adaptive denoising framework dynamically adjusts the thresholds based on the temporal variations of physiological signals. This block not only enhances noise suppression capabilities but also thoroughly preserves the critical physiological features required for downstream multi-modal human activity recognition.

#### 2.3.3. Data Normalization and Framing

**Figure 1:**
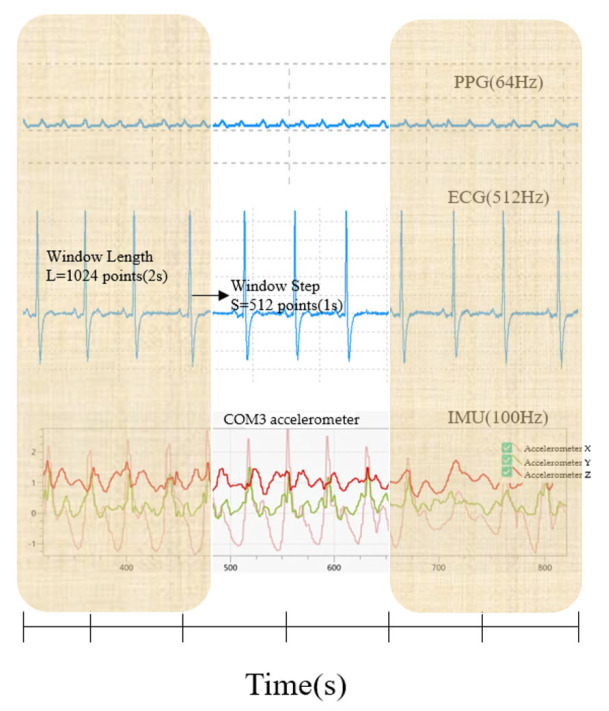
Schematic Diagram of Sliding Window Processing.

Due to the inconsistent sampling frequencies of motion and physiological sensors, synchronization of the multi-source heterogeneous signals is performed first. A linear interpolation algorithm is employed to align all signals to a common time base, followed by sample segmentation using a sliding window technique. The window length is set to 2 s with a step size of 1 s, resulting in a 50% overlap rate, as illustrated in Figure 1. To eliminate dimensional discrepancies, Z-score normalization is applied to all input data according to Equation (21):

(21) $$x^{\prime }=\frac{x-\mu }{\sigma }$$

where μ is the sample mean and σ is the sample standard deviation. A total of ten subjects were recruited in this study for dataset collection, with each subject completing five experimental sessions, each lasting approximately 10 min. The preprocessed data were subsequently framed using a sliding window approach with a window duration of 2 s and a stride of 1 s, segmenting the continuous multi-modal sensor data streams with a 50% overlap between adjacent windows. This framing strategy preserves the temporal continuity of movements while providing the model with dimensionally uniform and information-dense input tensors.

## 3. Attention-Based Two-Stream Deep Fusion Convolutional Neural Network

To address the limitations of traditional single-modal motion classification methods, such as low recognition rates in complex environments and insufficient perception of atypical gait patterns in visually impaired individuals, this paper proposes an Attention-based Two-Stream Deep Fusion Convolutional Neural Network. The model utilizes parallel branches to extract deep features from motion and physiological signals independently. Furthermore, an attention mechanism is introduced to achieve dynamic weighted fusion at the feature level.

### 3.1. Design of the Two-Stream Feature Extraction Network

**Figure 2:**
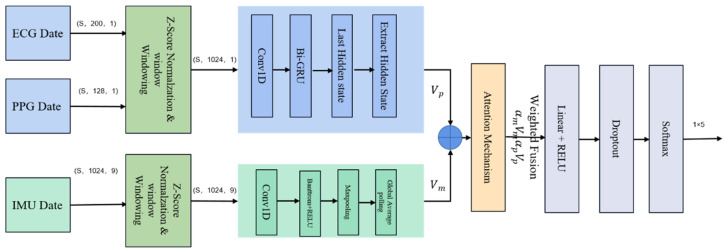
Architecture of the proposed Attention-based Two-Stream Deep Fusion Convolutional Neural Network.

The model adopts a dual-stream structure to extract features in parallel. The complete architecture of the Attention-based Two-Stream Deep Fusion Convolutional Neural Network is illustrated in Figure 2.

The model receives two parallel paths of heterogeneous signals at the input layer: the motion signal stream on the left, comprising tri-axial acceleration and tri-axial angular velocity, and the physiological signal stream on the right. This two-stream input design establishes the foundation for subsequent feature-level fusion.

In the feature extraction layer, the model employs differentiated structures tailored to the specific characteristics of each signal. The motion stream branch utilizes a 1D Convolutional Neural Network (1D-CNN). By stacking convolutional kernels, Batch Normalization (BN), ReLU activation, and max-pooling, it ultimately extracts a 128-dimensional motion feature vector $V_{m}$ through a Global Average Pooling (GAP) layer. Simultaneously, the physiological stream branch adopts a hybrid architecture combining a CNN with Bidirectional Gated Recurrent Units (Bi-GRU). This structure not only extracts morphological features but also explores the temporal dependencies of ECG and PPG signals through a dual-layer Bi-GRU, outputting a 64-dimensional physiological feature vector $V_{p}$.

The attention fusion layer aligns the extracted $V_{m}\;and\;V_{p}$ and automatically calculates the contribution coefficients, $\alpha_{m}$ and $\alpha_{p}$, via an attention weight allocator. Through this weighted fusion mechanism, the model generates a 192-dimensional deep fusion feature vector $V_{f}$. This allows the model to dynamically adjust its focus between physiological and motion signals based on current movement states, such as the moment of a fall.

The fusion feature layer consists of fully connected linear layers ReLU activation functions, and a Dropout layer (rate of 0.5) to prevent overfitting. The final output layer is a Softmax classification layer, which generates a probability distribution across five predefined activity categories: walking, standing, climbing stairs, descending stairs, and falling. This architecture enables effective motion intention recognition and behavior classification for the blindfolded mobility simulation. The specific architectural parameters of the proposed model are detailed in Table 1. The proposed ATS-DFCNN model was trained and evaluated on a workstation equipped with an Intel Core i7-12700K processor (Intel Corporation, Santa Clara, CA, USA) and an NVIDIA GeForce RTX 3060 graphics card (NVIDIA Corporation, Santa Clara, CA, USA).

#### 3.1.1. Preprocessing and Alignment of Heterogeneous Signals

The initial processing layer of the model addresses discrepancies in sampling rates and dimensions among multiple physical quantities. For PPG, ECG, and motion signals, the system employs a linear interpolation function S(t) to uniformly up-sample all signals to a reference frequency of 512 Hz. Subsequently, Z-score normalization is utilized to eliminate dimensional effects. The processed signals then enter a sliding window segmentation phase, yielding the input tensor $X_{in}$. The normalization formulas are defined in Equations (22) and (23):

(22) $$S(t)=a_{i}(t-t_{i}{)}^{3}+b_{i}(t-t_{i}{)}^{2}+c_{i}(t-t_{i})+d_{i}$$

(23) $$x^{\prime }=\frac{x-\mu }{\sigma }$$

In the equations above, μ and σ represent the sample mean and sample standard deviation within the sliding window, respectively. This step ensures that the subsequent gradient optimization of the model is performed within a stable manifold space.

#### 3.1.2. Two-Stream Heterogeneous Feature Extraction Network

The middle section of the model consists of two parallel processing branches, designed to handle “physical movement” and “physiological state”—two categories of features with distinct attributes.

(1) Motion Stream: A 1D Convolutional Neural Network (1D-CNN) is employed to operate directly on the tri-axial IMU signals. The convolutional layer encodes spatial features using sliding kernels W, extracting patterns of change in limb acceleration and angular velocity. The output of a single layer can be expressed as Equation (24):

(24) $$h_{motion}=f(W\times X_{IMU}+b)$$

(2) Physiological Stream: A hybrid CNN-GRU structure is utilized for this branch. First, the CNN extracts local morphological features from the ECG and PPG signals. These features are then fed into Bidirectional Gated Recurrent Units (Bi-GRU) to capture long-term dependencies of heart rate variations relative to motion states. The update logic for the hidden state $h_{t}$ is defined in Equation (25):

(25) $$h_{t}=GRU(x_{t},h_{t-1})$$

Ultimately, the two streams output highly generalized feature vectors: the motion feature vector $V_{m}$ and the physiological feature vector $V_{p}$.

#### 3.1.3. Attention Mechanism and Adaptive Feature Fusion

This section represents the core innovation of the proposed model. An attention mechanism is introduced to dynamically regulate the contribution of different modalities to the final decision. The system employs a compact fully connected network to calculate two weight parameters, $\alpha_{m}$ and $\alpha_{p}$, which represent the significance of the motion stream and the physiological stream, respectively. These weights are normalized via a Softmax function, as defined in Equation (26):

(26) $$\alpha_{i}=\frac{e^{Score_{i}}}{\sum e^{Score_{j}}},i\in \{m,p\}$$

The resulting comprehensive fusion feature vector $V_{f}$ is a weighted concatenation of the original feature vectors and their corresponding weights, as shown in Equation (27):

(27) $$V_{f}=\alpha_{m}\cdot V_{m}⊕\alpha_{p}\cdot V_{p}$$

In this context, ⊕ denotes the vector concatenation operator, while $\alpha_{m}$ and $\alpha_{p}$ are the weight coefficients automatically allocated by the model based on the current motion characteristics. This mechanism allows the model to mitigate noise interference in IMU signals caused by vigorous movements by increasing the physiological weight $\alpha_{p}$. By verifying the subject’s actual vital sign changes, the model achieves superior classification accuracy.

This mechanism enables the network to autonomously prioritize more informative modal features across diverse motion states and noisy environments. The attention weights, alongside all trainable parameters of the network, are automatically and iteratively updated via end-to-end backpropagation without any human-predefined weight coefficients; thus, the model can adaptively learn the significance of each modality directly from the training data.

#### 3.1.4. Deep Classifier and Output

The fused feature vector is fed into a classifier consisting of two fully connected layers. The final layer utilizes a Softmax function to output the probability distribution across five motion categories: walking, standing, climbing stairs, descending stairs, and falling. The cross-entropy loss function is employed for model training, as defined in Equation (28):

(28) $$L=-\sum_{i=1}^{N}\;\sum_{j=1}^{C}\;y_{i,j}log({\hat{y}}_{i,j})$$

In this equation, N denotes the number of samples, C represents the number of categories, y is the ground-truth label, and $\hat{y}$ is the predicted probability. The network parameters are iteratively optimized through the backpropagation algorithm to achieve the optimal classification accuracy.

## 4. Experiments and Results

### 4.1. Subjects and Equipment

**Figure 3:**
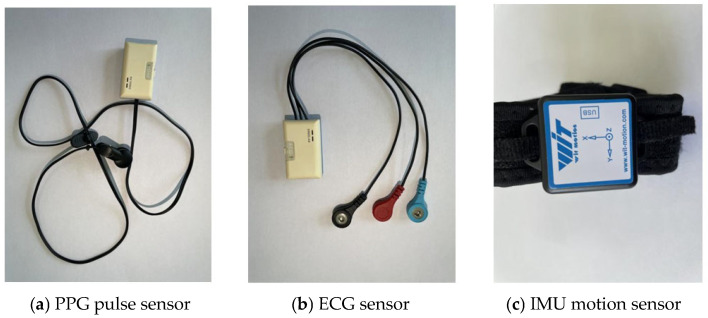
Experimental Setup.

To verify the effectiveness of the ATS-DFCNN model in motion recognition for the blindfolded healthy subjects, ten healthy volunteers were recruited to simulate blind motion states for data collection. Prior to the experiments, all subjects received training in white cane techniques and wore blindfolds throughout the sessions to simulate a visually impaired environment. The experimental setup included an IMU module integrating a tri-axial accelerometer and gyroscope (worn at the center of the waist), a portable single-lead ECG monitor (attached to the chest), and a flexible PPG pulse sensor (worn on the earlobe). All sensors transmitted data synchronously to a host computer via Bluetooth modules. The experimental equipment is illustrated in Figure 3, and the specifications of the sensors used in the simulation experiments are summarized in Table 2. The multi-modal kinematic and physiological signals were synchronously collected using the data acquisition system (Kingfar International Inc., Beijing, China).

### 4.2. Experimental Design and Data Collection

To ensure the diversity and reliability of the collected data, a standardized data collection protocol was developed. The experimental scenarios were conducted in indoor laboratories and simulated residential communities, ensuring that ground materials included various physical interfaces such as floor tiles, wooden flooring, and stairs. Regarding sensor deployment, subjects wore a perception belt equipped with an IMU module at the waist to capture center-of-gravity shifts. ECG electrodes were attached to the left chest, and a PPG photoelectric sensor was worn on the earlobe.

During the experiment, each subject completed five sets of cyclic movements according to predefined instructions:

(1) Walking and Standing: Subjects performed continuous movements for 2 min each in indoor corridors and stairwells to obtain stable gait patterns and physiological baseline data.
(2) Climbing and Descending Stairs: Operations were conducted in a standard residential stairwell for 2 min to extract feature differences arising from vertical displacement.
(3) Simulated Falls: To ensure subject safety, fall experiments were conducted on thickened foam mats. Subjects were required to simulate random behaviors, including forward falls, backward sitting falls, and lateral falls.

In this experiment, ten healthy participants were recruited to perform the blindfolded trials. To ensure the evaluation of cross-subject generalizability, a subject-independent validation protocol was adopted: data from eight participants were used for model training, one participant’s data served as the validation set, and the remaining participant’s data were reserved exclusively for the final independent testing.

Each participant completed five sessions of data collection, with each session lasting 10 min, yielding a total of 500 min of raw, continuous, multi-modal data streams. After preprocessing stages—which included baseline wander removal, truncation of idle intervals before and after movements, and elimination of severe artifacts—approximately 200 min of effective core signals were retained for algorithmic slicing. To guarantee experimental integrity and reproducibility, a single experimental sample was defined as a 2-s synchronized multi-channel data tensor extracted via a sliding window approach. Consequently, a total of 12,000 valid data samples were collected, establishing a comprehensive labeled benchmark dataset. Each sample not only captures physical spatial changes via acceleration and angular velocity but also preserves transient heart rate surges and pulsatile waveform fluctuations during action transitions, providing substantial data support for the subsequent attention mechanism to dynamically allocate weights between kinematic and physiological features.

### 4.3. Analysis of Experimental Results

**Figure 4:**
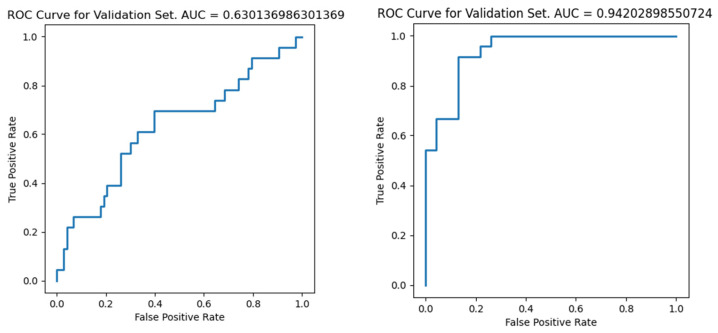
Comparative results before and after the algorithm improvement.

By analyzing the Receiver Operating Characteristic (ROC) curves on the validation set, we evaluate the significant performance enhancement brought by the LSTM-improved wavelet thresholding algorithm for subsequent feature extraction and classification. As shown in Figure 4, the Area Under the Curve (AUC) for the traditional unimproved wavelet thresholding denoising algorithm is only 0.630, indicating limited discriminability of the features extracted after denoising. Traditional fixed thresholds suffer from poor adaptability when processing complex multi-modal signals, which may result in the attenuation or distortion of valid signal features during the denoising process.

In contrast, the improved algorithm employing LSTM for adaptive threshold adjustment achieves a substantially higher AUC value of 0.942. The steepness of this curve at low False Positive Rates (FPR) signifies that the classifier possesses high sensitivity and promising discriminative power. These results demonstrate that the adaptive threshold learned via LSTM effectively balances noise suppression with the preservation of signal details. This provides more informative inputs for the subsequent classification model, ultimately leading to a marked improvement in overall classification performance.

Table 3 presents experimental results of the proposed ATS-DFCNN model under the subject-independent validation protocol. The empirical findings demonstrate that the model maintains promising and stable recognition performance even when evaluated on unseen subjects, achieving an average classification accuracy of 92.2%. The accuracy across individual folds ranges from 90.1% to 94.7%, exhibiting minimal performance fluctuations among different subjects. This indicates that the proposed model possesses excellent robustness and cross-subject generalizability, rather than merely relying on the motion patterns of specific individuals. These results further validate that the proposed multi-modal fusion architecture can effectively learn universal representations of kinematic and physiological features, thereby mitigating the risk of model overfitting to individual-specific characteristics.

**Figure 5:**
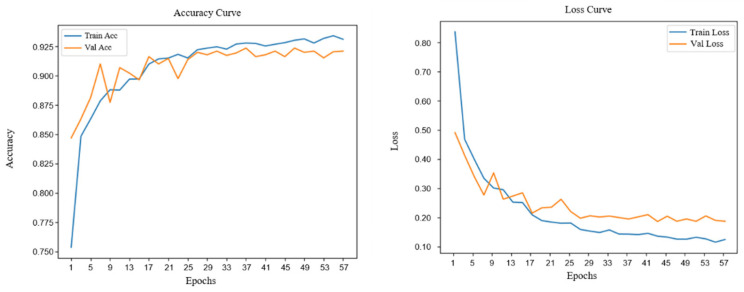
Experimental results of the proposed model.

This study utilizes ROC curves to present the classification results of the network model. The training and testing performances on the dataset are illustrated in the figures below. The model was trained for 60 epochs, with both accuracy and loss beginning to stabilize around the 35th epoch. Figure 5 displays the accuracy–epoch curve (left) and the loss–epoch curve (right), demonstrating the convergence characteristics of the network.

**Figure 6:**
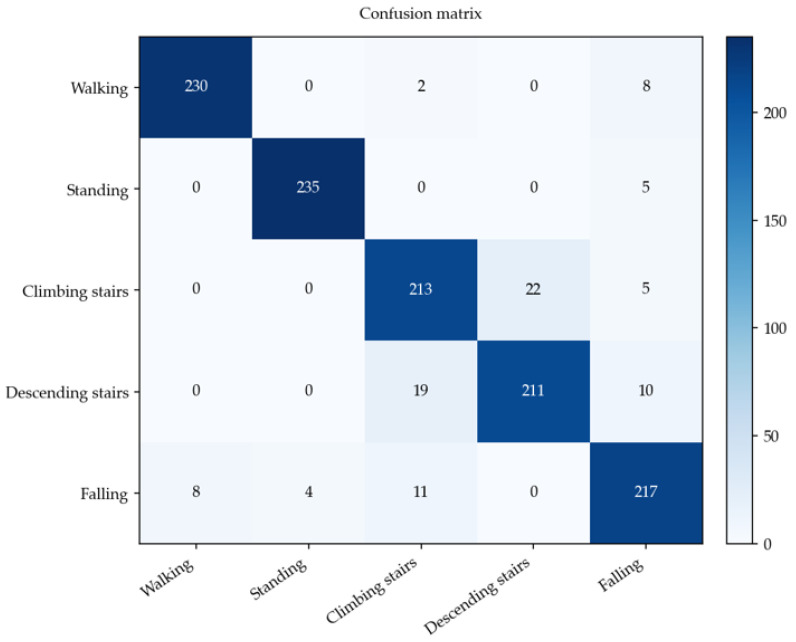
Confusion matrix.

Figure 6 presents the confusion matrix of the proposed model. It can be clearly observed that the model achieves a higher recognition rate for the “standing” category, whereas the classification accuracies for “climbing/descending stairs” and “falling” are relatively lower.

As indicated in Table 4, the system achieves an average recognition accuracy of 92.2% across the five movement categories for the blindfolded healthy subjects. Specifically, the categories of “standing” and “walking” exhibit the highest recognition rates, both exceeding 97%. For the most challenging “falling” behavior, the accuracy reaches 92.1%. Compared to traditional detection methods relying solely on motion signals, the false alarm rate is reduced by 11.2%.

### 4.4. Ablation Study

To further verify the contributions of multi-source data fusion and the attention mechanism to feature extraction in complex environments, a set of ablation experiments was conducted. As shown in Table 5, a comparative analysis was performed between the proposed ATS-DFCNN model and traditional convolutional neural networks along with their variants. The results indicate that the proposed model outperforms all baseline models across all classification metrics. Compared to the baseline CNN model, the recognition accuracy of our method increased by 4.8%. This significant performance enhancement is primarily attributed to the complementary feature extraction capability of the two-stream deep fusion architecture for heterogeneous signals. Furthermore, the introduction of the attention mechanism enables dynamic allocation of feature weights, which further reinforces key motion characteristics and improves classification precision.

### 4.5. Comparative Experiments

To verify the effectiveness of the ATS-DFCNN framework, comparative experiments were conducted against several representative state-of-the-art models in the field of temporal human activity recognition, including the one-dimensional residual network (1D-ResNet), the convolutional long short-term memory network (CNN-LSTM), and the one-dimensional vision Transformer (ViT-1D). As indicated by the results in Table 6, the proposed ATS-DFCNN model achieved the optimal overall recognition accuracy among all benchmarking methods. These experimental results demonstrate that integrating multi-modal physiological and kinematic information, combined with an adaptive attention fusion mechanism, can significantly enhance the feature discriminability in complex human activity recognition tasks.

To further investigate the impact of multi-channel data fusion and the attention mechanism, a series of comparative experiments were conducted. As summarized in Table 7, the recognition accuracy using the single IMU modality is 87.4%. With the incorporation of ECG signals, the accuracy improves to 90.1%, and further rises to 91.2% upon integrating PPG signals. This progression demonstrates the compensatory role of physiological feedback in distinguishing between similar movements, such as rapid squatting and falling. Ultimately, by introducing the Attention Mechanism, the system becomes capable of dynamically adjusting weights based on signal quality, further optimizing the recognition rate to 92.2%. These results indicate that the ATS-DFCNN model effectively integrates heterogeneous data features, offering significant advantages in ensuring both the real-time performance and accuracy of motion monitoring for the blindfolded healthy subjects.

## 5. Conclusions

In response to the requirements for behavioral monitoring and safety early-warning for the blindfolded healthy subjects in complex environments, this study proposes and designs an Attention-based Two-Stream Deep Fusion Convolutional Neural Network (ATS-DFCNN) model. By integrating tri-axial accelerometers, gyroscopes, ECG, and PPG sensors, we constructed a multi-modal motion and physiological signal acquisition system. This approach effectively addresses the limitations of traditional unimodal detection schemes, such as low recognition rates and high false-alarm frequencies under complex gait patterns.

Experimental results demonstrate that the proposed two-stream architecture can concurrently extract physical spatial features of limb movements and physiological temporal features of human stress responses. By introducing a feature-level attention mechanism, the model achieves dynamic weight adjustment for heterogeneous signals, exhibiting promising discriminative power when detecting sudden hazardous events like “falls.” This significantly reduces the probability of misclassifying daily activities as danger signals. Testing results indicate that the system achieves an average recognition accuracy of 92.2% across five typical actions—walking, standing, climbing/descending stairs, and falling—representing a 4.8% improvement over the single IMU modality and validating the superiority of multi-modal fusion in assistive monitoring.

Nevertheless, because this study is in its validation stage rather than a clinical deployment phase, the experiments were conducted using trained, healthy participants in a blindfolded manner, and no clinically diagnosed visually impaired individuals were recruited. Consequently, the current findings can only demonstrate the feasibility of the proposed framework under controlled experimental conditions. Future research will expand the sample size and recruit participants with varying degrees of visual impairment to further enhance the clinical applicability and generalizability of the system.

Although the proposed ATS-DFCNN framework exhibits excellent multi-modal human activity recognition capabilities, model explainability and decision trustworthiness remain critical challenges to be addressed in biomedical assistive system applications. By leveraging the attention fusion mechanism, the model can dynamically reflect the weight proportions of physiological signals and kinematic modalities during the classification process, thereby demonstrating a certain degree of inherent interpretability.

However, a comprehensive explainable artificial intelligence (XAI) framework has not yet been integrated into this study. Future research will explore explainable multi-modal learning methodologies, including attention visualization, SHAP-based (SHapley Additive exPlanations) feature attribution analysis, gradient-weighted class activation mapping (Grad-CAM) visualization, and uncertainty-aware confidence estimation techniques. These methods can effectively elevate model decision transparency and result credibility, further enhancing the user trust of visually impaired individuals utilizing wearable assistive devices.

In conclusion, the research work presented in this study not only provides a effective, highly promising algorithmic architecture for motion intent inference for the blind but also offers a valuable technical reference for multi-source physiological information fusion in wearable health monitoring devices. Future studies will focus on optimizing the lightweight architecture of the model, aiming to deploy the algorithm onto embedded mobile terminals. Combined with real-time tactile feedback technology, the framework proposed in this study exhibits significant potential for application in future research on multi-modal wearable assistive technologies.

## Figures and Tables

**Table 1:** Specific hyperparameter and structural configurations of the proposed network.

Parameter	Value	Parameter	Value
IMU stream layers	3-layer CNN (Kernels: 5, 3, 3)	Optimizer	Adam
ECG/PPG Stream	2-layer CNN + LSTM	Learning Rate	1 × 10^−4^
Activation	ReLU	Batch Size	32
Training Time	60 epochs	Hardware	Intel i7-12700K, NVIDIA RTX 3060

**Table 2:** Sensor parameters for the simulation experiments.

Sensor Name	Signal Category	Sampling Rate	Application
ErgoLAB Wireless PPG Sensor	Pulse (PPG) signal	64 Hz	Physiological signal analysis
ErgoLAB Wireless ECG Sensor	ECG signal	512 Hz	Physiological signal analysis
BWT61CL IMU Sensor	Motion (Inertial) signal	100 Hz	Motion signal analysis

**Table 3:** Performance of the proposed ATS-DFCNN model under the subject-independent validation protocol.

Subject ID	Accuracy	Recall	F1-Score
S1	92.1%	91.8%	91.9%
S2	94.1%	93.7%	93.9%
S3	93.2%	92.2%	93.0%
S4	90.1%	89.9%	89.9%
S5	90.9%	90.5%	90.7%
S6	89.4%	89.0%	89.2%
S7	92.3%	91.9%	92.1%
S8	94.7%	94.2%	94.4%
S9	93.0%	92.6%	92.8%
Average	92.2%	91.8%	92.0%

**Table 4:** Performance evaluation metrics for different motion categories.

Activity Category	Precision	Recall	F1-Score
Walking	96.5%	97.2%	96.8%
Standing	98.2%	99.0%	98.6%
Climbing stairs	91.8%	90.5%	91.1%
Descending stairs	90.6%	89.4%	90.0%
Falling	92.3%	91.8%	92.0%

**Table 5:** Comparative results of the ablation study.

Model Architecture	Accuracy	Recall	F1-Score
CNN	87.4%	87.2%	87.3%
CNN-GRU	89.7%	90.0%	89.9%
CNN-Attention	91.8%	91.5%	91.6%
Proposed (ATS-DFCNN)	92.2%	91.8%	92.0%

**Table 6:** Performance comparison between the proposed ATS-DFCNN and other state-of-the-art architectures for human activity recognition.

Model	Accuracy	Recall	F1-Score
CNN-LSTM	89.8%	89.0%	0.89
1D-ResNet	90.7%	91.0%	0.90
ViT-1D	91.5%	91.2%	0.91
ATS-DFCNN	92.2%	92.4%	0.92

**Table 7:** Comparative analysis of different sensing modalities.

Sensing Modalities	With/Without Attention	Accuracy
IMU	✕	87.4%
IMU + ECG	✕	90.1%
IMU + ECG + PPG	✕	91.2%
IMU + ECG + PPG	√	92.2%

## Data Availability

The relevant codes of this article have been sorted out. The code logic is clear and the structure is reasonable. The dataset is a self-collected dataset, and we do not plan to make it public.
